# Immunisation status of UK-bound refugees between January, 2018, and October, 2019: a retrospective, population-based cross-sectional study

**DOI:** 10.1016/S2468-2667(22)00089-5

**Published:** 2022-05-28

**Authors:** Anna Deal, Sally E Hayward, Alison F Crawshaw, Lucy P Goldsmith, Charles Hui, Warren Dalal, Fatima Wurie, Mary-Ann Bautista, May Antonnette Lebanan, Sweetmavourneen Agan, Farah Amin Hassan, Kolitha Wickramage, Ines Campos-Matos, Sally Hargreaves

**Affiliations:** a Migrant Health Research Group, Institute for Infection and Immunity, St George's, University of London, London, UK; b Faculty of Public Health and Policy, London School of Hygiene & Tropical Medicine, London, UK; c Department of Paediatrics, University of Ottawa, ON, Canada; d International Organization for Migration, Nairobi, Kenya; e International Organization for Migration, Manila, Philippines; f Office for Health Improvement and Disparities, Department of Health and Social Care, London, UK

## Abstract

**Background:**

WHO's new Immunization Agenda 2030 places a focus on ensuring migrants and other marginalised groups are offered catch-up vaccinations across the life-course. Yet, it is not known to what extent specific groups, such as refugees, are immunised according to host country schedules, and the implications for policy and practice. We aimed to assess the immunisation coverage of UK-bound refugees undergoing International Organization for Migration (IOM) health assessments through UK resettlement schemes, and calculate risk factors for under-immunisation.

**Methods:**

We undertook a retrospective cross-sectional study of all refugees (children <10 years, adolescents aged 10–19 years, and adults >19 years) in the UK resettlement programme who had at least one migration health assessment conducted by IOM between Jan 1, 2018 and Oct 31, 2019, across 18 countries. Individuals' recorded vaccine coverage was calculated and compared with the UK immunisation schedule and the UK Refugee Technical Instructions. We carried out multivariate logistic regression analyses to assess factors associated with varying immunisation coverage.

**Findings:**

Our study included 12 526 refugees of 36 nationalities (median age 17 years [IQR 7–33]; 6147 [49·1%] female; 7955 [63·5%] Syrian nationals). 26 118 vaccine doses were administered by the IOM (most commonly measles, mumps, and rubella [8741 doses]). During the study, 6870 refugees departed for the UK, of whom 5556 (80·9%) had at least one recorded dose of measles-containing vaccine and 5798 (84·4%) had at least one dose of polio vaccine, as per the UK Refugee Technical Instructions, and 1315 (19·1%) had at least one recorded dose of diphtheria-containing vaccine or tetanus-containing vaccine. 764 (11·1%) of refugees were fully aligned with the UK schedule for polio, compared with 2338 (34·0%) for measles and 380 (5·5%) for diphtheria and tetanus. Adults were significantly less likely than children to be in line with the UK immunisation schedule for polio (odds ratio 0·0013, 95% CI 0·0001–0·0052) and measles (0·29, 0·25–0·32).

**Interpretation:**

On arrival to the UK, refugees' recorded vaccination coverage is suboptimal and varies by age, nationality, country of health assessment, and by disease, with particularly low coverage reported for diphtheria and tetanus, and among adult refugees. These findings have important implications for the delivery of refugee pre-entry health assessments and catch-up vaccination policy and delivery targeting child, adolescent, and adults migrants in the UK, and other refugee-receiving countries. This research highlights the need for improved data sharing and clearer definition of where responsibilities lie between host countries and health assessment providers.

**Funding:**

UK National Institute for Health Research (NIHR300072) and Medical Research Council (MR/N013638/1).

## Introduction

The scale of international migration has increased substantially in the past two decades. In 2020, the number of international migrants (defined as people residing outside their country of birth or usual country of residence)^1^ globally was estimated to be 272 million, or 3·5% of the world's population, of whom nearly two-thirds were labour migrants.^2^

Forcible displacement is also on the increase due to persecution and violence, such as the Rohingya forced to seek safety in Bangladesh, the continuing conflicts in Syria, Democratic Republic of the Congo, and South Sudan, and climate-related disasters.^2^

In 2019, the global refugee population reached 26 million,^3^ of whom less than 1% are resettled each year.^4^ Refugees—defined as people from outside their country of origin due to persecution, conflict, generalised violence, or similar^1^—from countries with disrupted health systems could be at risk of being undervaccinated,^5^, ^6^ and outbreaks of vaccine-preventable disease such as measles, diphtheria, and hepatitis A have been documented in refugee populations in Europe in the past two decades.^7^, ^8^, ^9^ A systematic review has highlighted that country of origin, recent migration, and refugee or asylum status were important determinants of underimmunisation among migrants in Europe.^10^ During the COVID-19 pandemic, refugee and migrant populations are known to have had increased risk of infection and potentially worse adverse outcomes than the general population.^11^ Despite this finding, low COVID-19 vaccination uptake and intent has been shown in migrant populations in the few countries where this has been measured,^12^, ^13^, ^14^ highlighting the importance of ensuring equitable access to vaccination systems for marginalised populations.

**Research in context**

**Evidence before this study**

Migrants, including forced migrants such as refugees, could be an undervaccinated group globally and face barriers to accessing vaccination systems and catch-up vaccines. A systematic review by Mipatrini and colleagues has previously shown that migrants to Europe are underimmunised for several key vaccine-preventable diseases; another study by Deal and colleagues has shown that outbreaks of vaccine-preventable diseases have occurred among refugees and migrants residing in temporary camps or reception centres in the European context, yet these groups could have disproportionate difficulty accessing vaccination services. A systematic review by Crawshaw and colleagues found country of origin, being a recently arrived migrant, or a refugee or asylum seeker, were frequently found to be statistically significant determinants of underimmunisation for migrants to European countries. A Europe-wide survey of experts and a policy analysis found a shortage of systems in place in most European countries to identify undervaccinated refugees and migrants, in particular adolescent and adult refugees and migrants, and offer them catch-up vaccination for vaccines they could have missed as children, missed doses, or additional vaccines to align them with their host country schedule. In its new Immunization Agenda 2030, WHO has called for greater focus to be placed on vaccination across the life-course, including a greater focus on vulnerable groups including migrants to ensure equitable access to routine vaccination. However, little is known to date about the immunisation coverage for key vaccine-preventable diseases among specific migrant groups, or the implications this will have for vaccination services and health systems on arrival to their host country.

**Added value of this study**

This retrospective cross-sectional analysis explored for the first time the immunisation coverage of a specific group of migrants—refugees within a formal resettlement programme—being resettled in the UK. Our results show that more than one in ten refugees depart for the UK with no recorded polio-containing vaccine, and almost one in five with no recorded measles-containing vaccines, suggesting they will require catch-up vaccination services on arrival in the UK. We found adult and adolescent refugees are less likely to have recorded immunisations for key vaccines compared with children on departure to the UK, suggesting targeted initiatives on arrival to the UK are warranted. New approaches could be urgently considered and developed for service delivery and planning for vaccination systems in the UK and other refugee-receiving countries. This study also highlights major shortfalls in data collection and the need for more robust mechanisms to assess under-immunisation in refugees and strengthen data flows between International Organization for Migration and other non-governmental organisations delivering vaccines in humanitarian contexts, and refugee-receiving countries.

**Implications of all the available evidence**

This research has far-reaching implications for all refugee-receiving countries, including the UK, as it shows that large numbers of refugees could be arriving to host countries underimmunised. Of particular concern is that adult refugees are most likely to have no previous recorded vaccination, but it is these who are often excluded from vaccination services on arrival, with no systems in place in many countries to offer catch-up vaccination for vaccinations missed in their home country, or to align them with the host country schedule. Although these data relate specifically to refugee populations, our results are likely to have implications regarding the expected vaccination coverage of other migrant groups arriving from similar countries and regions of origin. Pre-entry health assessments and domestic initiatives have the potential to improve vaccination coverage among refugees and other migrant groups—a key objective of the new WHO Immunization Agenda 2030 framework for action. The extent to which barriers to vaccination systems and vaccine hesitancy play a role in low levels of vaccination merits further exploration.

Many receiving countries have official resettlement and health assessment programmes for refugees, including the USA, Canada, Australia, New Zealand, and the UK.^15^, ^16^, ^17^ In 2004, the UK Government launched the Gateway Protection Programme to help particularly vulnerable refugees wanting to settle in the UK,^17^, ^18^ followed by three further resettlement schemes including the Vulnerable Persons Resettlement Programme Scheme and the Vulnerable Children's Resettlement Scheme, both created in response to the Syrian conflict,^19^, ^20^ and the Family Reunion Travel Assistance Programme. All four of these resettlement schemes are hereafter referred to collectively as the UK resettlement programme. The Vulnerable Persons Resettlement Programme Scheme was the most high-profile UK scheme; 20 319 individuals were resettled under the scheme before its end in February, 2021, when all schemes were combined to form the new UK Resettlement Scheme.^17^, ^21^ Under the UK resettlement programme, refugees were resettled to the UK following a detailed pre-entry health assessment, to which all refugees eligible for resettlement are entitled.

For more than 65 years, the International Organization for Migration (IOM) has played a vital role in resettlement operations globally, carrying out migration health assessments for refugees before resettlement.^22^, ^23^ A key part of the International Organization for Migration's health assessments for refugees includes documenting immunisation history and administering key vaccines to protect them from vaccine-preventable diseases and to sustain herd immunity in host countries.^24^ As part of its global programmes, the International Organization for Migration had provided more than 445 000 vaccine doses by June, 2020, to around 141 000 individuals across 80 countries during pre-departure migration health assessments.^24^

The UK Refugee Technical Instructions define the UK-specific guidelines for vaccination of refugees during the migration health assessments (appendix p 1).^25^ The UK Refugee Technical Instructions also state that “where possible, the UK immunisation algorithm for vaccination of individuals with uncertain or incomplete immunisation status shall be followed”, which prioritises vaccination with two doses of measles, mumps, and rubella (MMR), three doses of tetanus-diphtheria/inactivated polio vaccine (polio), and *Neisseria meningiditis* B, C, and ACWY conjugate vaccines (MenACWY; for individuals aged 10–25 years), and advises that individuals with missing immunisation records should be treated as unvaccinated and re-administered doses.^26^, ^27^ Historically in the UK, data for immunisation status of refugees and migrants have been poor as migrant status is not routinely collected in the UK's National Health Service (NHS), meaning it is difficult to target catch-up vaccination for those who are most in need.

We did a retrospective population-based cross-sectional study using data from the IOM health assessments to assess vaccinations administered, immunisation history on departure to the UK, and alignment of resettled refugees with the UK Refugee Technical Instructions, and the UK's immunisation schedule and investigated factors potentially associated with differing immunisation history. This study will inform several strands of health policy, including strengthening refugee pre-entry health assessments and domestic initiatives to improve catch-up vaccination and immunisation coverage among resettled refugees entering the UK and other high-income countries, aligning with WHO's Immunization Agenda 2030 framework for action.

## Methods

### Study design and participants

We undertook a retrospective cross-sectional study of demographics, vaccine records, and health data of all UK-bound refugees undergoing routine pre-departure International Organization for Migration health assessments between Jan 1, 2018, and Oct 31, 2019. All refugees resettling to the UK undergo at least one premigration health check, as required under UK policy; however, vaccinations given during these health checks are not compulsory. The reporting of this study conforms to STROBE guidelines.

As part of the IOM health assessment process, applicants were given a full explanation of the health assessment, consent process, and how their data would be used by relevant UK authorities and agencies to guide service improvement. Consent was collected and all data fully anonymised before the research study took place; individuals were only identifiable by a randomly generated ID variable. All individuals were provided copies of their health assessment record.

As part of the International Organization for Migration health assessment process, applicants were given a full explanation of the health assessment, consent process, and how their data would be used by relevant UK authorities and agencies to guide service improvement. Consent was collected and all data fully anonymised before the research study took place. All individuals were provided copies of their health assessment record.

### Data sources

Data were collected from all IOM clinics enrolled in the UK pre-entry migration health assessments in 18 countries. Health data of all International Organization for Migration-assisted refugees were entered by the attending physician or nurse in the Migrant Management Operational System Application (MiMOSA), which is an IOM data management software.^28^ MiMOSA data are aggregated and undergo quality control in a central data repository. IOM regularly validates health assessment data using automated checks to verify and correct inconsistencies, in coordination with focal persons at the country level. Anonymised raw data was exported from the MiMOSA system and stored by the research team as password-protected.csv files, until the end of the project.

### Data characteristics and processing

Each entry in the database represents one migration health assessment and contains demographic data, medical history, and vaccinations given. The validity of a completed migration health assessment lasts for 1 year; however, refugees might be required to repeat migration health assessments if requested by the UK Home Office or a clinician. Multiple migration health assessments are distinguishable by the health assessment description variable, which records the chronological order of an individual's migration health assessment. For an individual who had departed to the UK, their final pre-departure health assessment visit is marked as departed. Unique ID codes for entries identified as twins were re-coded to represent unique individuals. For true duplicate entries, one of each pair was removed.

Vaccinations recorded include both those given during and before the assessment in question; prior vaccine doses include those given at previous assessments and given externally (ie, those recorded by the IOM on presentation of vaccination records). Vaccinations given before the migration health assessment in question are distinguished as historical doses.

Explanatory variables (ie, nationality [country of passport, or of birth in which no passport available], age, sex, and country of health assessment) were coded as categorical and dichotomous variables. Age was coded in three levels as per WHO definitions: children younger than 10 years, adolescents aged 10–19 years, and adults older than 19 years. The eight most commonly occurring nationalities were coded in a nationality variable. The countries of health assessment were classified into five WHO regions (Eastern Mediterranean, Europe, Africa, Asia, and Pacific).

### Statistical analysis

Data cleaning and analyses were carried out using R (version 3.2.1). All tests were two-tailed and p values less than 0·05 regarded as significant. Data analysis was undertaken in several steps. We first calculated total counts of vaccine doses (for each vaccine type) administered across the time period and classified by country of migration health assessment to analyse variation in vaccination activities by IOM site.

Two subsets of the database were created for further analysis; one containing data from the most recent migration health assessment for every individual, and the other only containing data from individuals who had already departed for the UK by the study end.

Using the subset containing all individuals, we described the demographics of refugees undergoing migration health assessments, and summarised continuous data using mean (SD) or median (IQR). For analyses on individual vaccine history, the subset of the database containing individuals who had already departed was used as this was considered the endpoint of the study. Individual vaccine history was calculated for each disease by combining historical doses with those given in the final migration health assessment (eg, for measles, all MMR and measles monovalent doses were combined). The proportion of refugees administered with no doses, at least one dose, and being vaccinated in line with the UK Refugee Technical Instructions (specific UK Refugee Technical Instructions guidelines exist for polio and measles only defined as at least one dose [appendix p 1]) and the UK immunisation schedule were calculated by disease. Being caught up to the UK immunisation schedule was defined as having the minimum number of doses required for their age (appendix p 1).^26^

For polio and measles, multivariate logistic regressions were done to calculate the odds ratios and associated 95% CIs for having at least one dose (as per the UK Refugee Technical Instructions) or being in line with the UK immunisation schedule, by sex, age, nationality, and country of application. For nationality (Syrian) and region (Eastern Mediterranean) of health assessment calculations, the group with the largest population were used as the reference group.

### Role of the funding source

The funders of the study had no role in study design, data collection, data analysis, data interpretation, or writing of the report.

## Results

**Figure 1:**
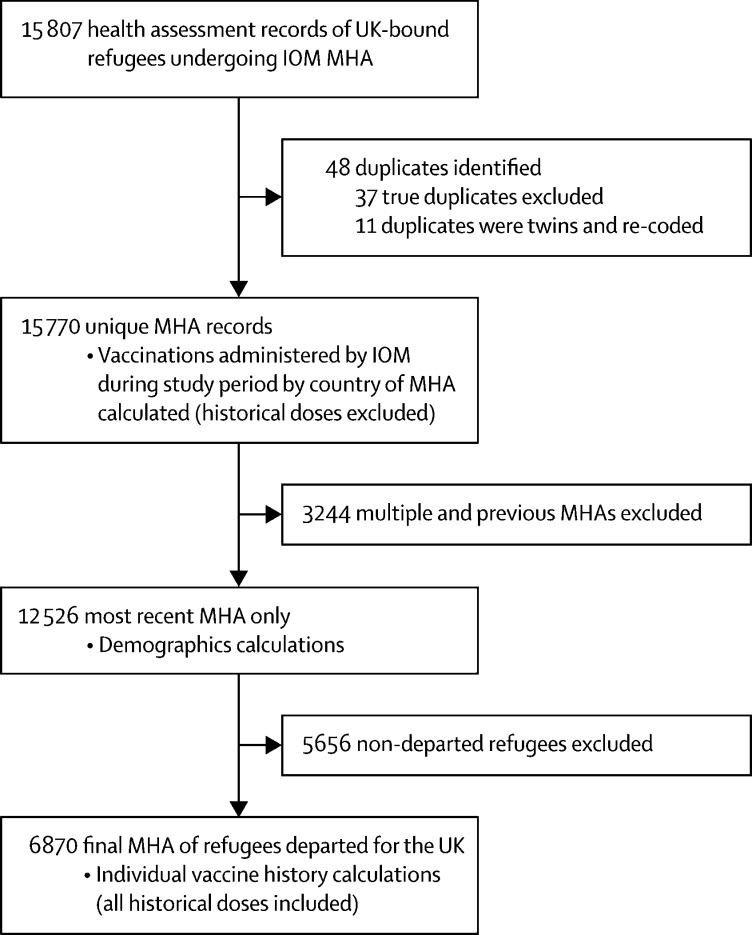
Study selection and analytical steps IOM=International Organization for Migration. MHA=Migration Health Assessment.

**Figure 2:**
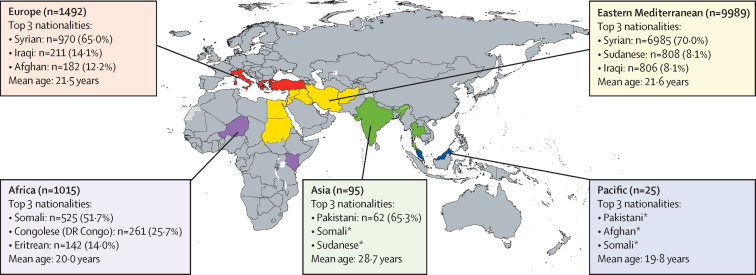
Demographic data of included UK-bound refugees by region of health assessment, January, 2018–October, 2019 (n=12 526) Coloured countries are those hosting migrant health assessments. *Small numbers suppressed for anonymity purposes.

12 526 individuals of 36 nationalities had at least one IOM health assessment visit as part of the UK resettlement programme between Jan 1, 2018 and Oct 31, 2019 (15 770 health assessments in total), with assessments taking place in 18 countries (Figure 1, Figure 2). There were no missing data for compulsory variables (ID numbers, age, nationality, or country of health assessment), therefore, no entries were excluded on these grounds.

3918 (31·3%) of 12 526 individuals were children (<10 years old), 2732 (21·8%) were adolescents (10–19 years old), and 5876 (46·9%) were adults (>19 years old). The median age of individuals in the database was 17 years (IQR 7–33). Demographics of departed refugees were similar to the individuals who had not yet departed by the end of the study (table 1).

**Table 1:** Demographics of UK-bound refugees who underwent an International Organization for Migration health assessment, January 2018–October 2019

		**All refugees assessed (n=12 526)**	**Refugees departed to the UK by study end (n=6870)**	**Refugees not departed by study end (n=5656)**
Age (years)		17 (IQ25:7, IQ75:33)	18 (IQ25:7, IQ75:33)	18 (IQ25:8, IQ75:33)
Age group				
	Child (<10 years)	3918 (31·3%)	2195 (32·0%)	1723 (30·4%)
	Adolescent (10–19 years)	2732 (21·8%)	1438 (20·9%)	1294 (22·9%)
	Adult (>19 years)	5876 (46·9%)	3237 (48·6%)	2639 (46·7%)
Sex				
	Female	6147 (49·1%)	3337 (48·8%)	2810 (49·7%)
	Male	6379 (50·9%)	3533 (51·2%)	2846 (50·3%)
Nationality				
	Syrian	7955 (63·5%)	5032 (73·2%)	2923 (51·7%)
	Iraqi	1018 (8·1%)	353 (5·1%)	665 (11·8%)
	Sudanese	821 (6·6%)	292 (4·3%)	529 (9·4%)
	Afghan	673 (5·4%)	155 (2·3%)	518 (9·2%)
	Somalian	664 (5·3%)	287 (4·2%)	377 (6·7%)
	Palestinian	411 (3·3%)	144 (2·1%)	267 (4·7%)
	Eritrean	268 (2·1%)	156 (2·3%)	112 (2·0%)
	Congolese (DR Congo)	262 (2·1%)	228 (3·3%)	34 (0·6%)
	Others (n=28)	454 (3·6%)	223 (3·2%)	231 (4·1%)
Region and country of health assessment				
Eastern Mediterranean		9899 (79·0%)	5561 (80·9%)	4338 (76·7%)
	Lebanon	3069 (24·5%)	1986 (28·9%)	1083 (19·1%)
	Jordan	2668 (21·3%)	1618 (23·6%)	1050 (18·6%)
	Egypt	2301 (18·4%)	1220 (17·8%)	1081 (19·1%)
	Iraq	1150 (9·2%)	631 (9·2%)	519 (9·2%)
	Syria	267 (2·1%)	..	..
	Afghanistan	210 (1·7%)	0	210 (3·7%)
	Iran	201 (1·6%)	72 (1·0%)	129 (2·3%)
	Sudan	33 (0·3%)	33 (0·5%)	0 (0)
Europe		1492 (11·9%)	669 (9·3%)	823 (14·6%)
	Turkey	1346 (10·7%)	556 (8·1%)	790 (1·4%)
	Greece	119 (1·0%)	86 (1·3%)	33 (0·6%)
	Italy	27 (0·2%)	27 (0·4%)	0 (0)
Africa		1015 (8·1%)	608 (8·9)	407 (7·2)
	Kenya	604 (4·8%)	310 (4·5%)	294 (5·2%)
	Burundi	243 (1·9%)	213 (3·1%)	30 (0·5%)
	Niger	168 (1·3%)	85 (1·3%)	83 (1·5%)
Asia		95 (0·8%)	15 (0·2%)	80 (1·4%)
	Sri Lanka	41 (0·3%)	0	41 (0·7%)
	Thailand	29 (0·2%)	15 (0·2%)	14 (0·2%)
	India	25 (0·2%)	0	25 (0·4%)
Pacific		25 (0·2%)	17 (0·2%)	8 (0·1%)
	Malaysia	25 (0·2%)	17 (0·2%)	8 (0·1%)

Data are median (IQR) or n (%). Country of health assessment not necessarily same as nationality. Small numbers (<5) suppressed for anonymity purposes, as shown by ..

9899 (79·0%) of refugees were assessed in the WHO Eastern Mediterranean region; mostly Lebanon (n=3069), Jordan (n=2668), and Egypt (n=2301; table 1). 36 nationalities were recorded, with the most frequent being Syrian (7955 [63·5%] of 12 526). 6985 (87·8%) of 7955 Syrians were assessed in the Eastern Mediterranean region and 970 (12·2%) were assessed in European centres. 12 305 (98·2%) of 12 526 refugees were in a country different from their nationality when undergoing their migration health assessments (figure 2).

During the study period 15 770 migration health assessments were carried out, and at least one vaccine dose was given in 12 344 [78·3%] assessments, with 26 118 vaccine doses administered in total. Most doses were given in Lebanon (8207 [31·4%] of 26 118) and Turkey (5822 [22·3%]. 7768 (29·7%) of the 26 118 doses were given to children, 7776 (29·8%) to adolescents, and 10 574 (40·5%) to adults. The vaccine most frequently given was MMR (8741 doses [33·5%] of 26 118), followed by oral polio vaccine (8251 doses [31·6%]), hepatitis B (4529 doses [17·3%]), inactivated polio vaccine (1549 doses [5·9%]), meningococcal vaccines (1429 doses [5·5%]), and diphtheria, tetanus, and pertussis (1143 doses [4·4%]; appendix pp 3–4).

**Figure 3:**
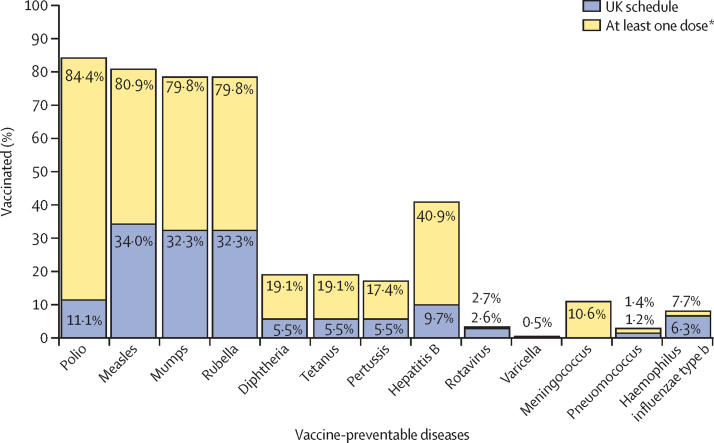
Vaccination coverage of UK-bound refugees with departed status at pre-departure checks for 13 key diseases, January 2018–October 2019 (n=6870) *For polio and measles, at least one dose is required to align to the UK Refugee Technical Instructions.

Among 6870 individuals who had already departed for the UK, immunisation coverages were calculated based on the International Organization for Migration-given and recorded historical doses. The proportions with at least one recorded dose (according to the UK Refugee Technical Instructions for polio and measles) and the UK immunisation schedule are shown in figure 3. For polio, most individuals (5798 [84·4%] of 6870) were recorded as being vaccinated according to the UK Refugee Technical Instructions guidelines and only 764 (11·1%) for the UK immunisation schedule. On measles coverage, 5556 (80·9%) of 6870 refugees were aligned with the UK Refugee Technical Instructions and 2338 (34·0%) with the UK immunisation schedule. Considerably lower diphtheria, tetanus, and pertussis coverage was reported; 1315 (19·1%) individuals had received at least one dose for diphtheria and tetanus before departure for the UK, and 380 (5·5%) were fully aligned to the UK immunisation schedule (figure 3; appendix pp 5–6).

726 (10·6%) of 6870 individuals had received at least one dose of meningococcal vaccines. 2809 (40·9%) had at least one dose of hepatitis B-containing vaccine, with 668 (9·7%) in line with the UK immunisation schedule. Few refugees (527 [7·7%] of 6870) had at least one *Haemophilus influenzae*-containing vaccine, with 430 (6·3%) in line with the UK immunisation schedule. 32 (0·5%) of 6870 had at least one recorded dose of varicella and 98 (1·4%) for pneumococcal vaccine (figure 3).

For polio, the percentage of child refugees with at least one recorded dose of vaccine, as per the UK Refugee Technical Instructions, was higher (1936 [88·2%] of 2195) than it was for adolescents (1190 [82·8%] of 1438) or adult refugees (2672 [82·5%] of 3237; table 2). There was a small difference in the proportion of adult (2637 [81·5%] of 3237), adolescent (1181 [82·1%] of 1438), and child (1738 [79.2%] of 2195) refugees aligned with the UK Refugee Technical Instructions for measles (at least one dose; table 3). For diphtheria/tetanus, only 316 (9·8%) adults had at least one recorded dose of vaccine, compared with 241 (16·8%) adolescents and 758 (34·5%) children. 1717 (78·2%) child and 1000 (69·5%) adolescent refugees had one recorded dose of hepatitis B vaccine, whereas this was only true for 92 (2·8%) of adults (appendix pp 5–6).

**Table 2:** Factors associated with being in line with the UK Refugee Technical Instructions (at least one dose) and being in accordance with the UK immunisation schedule for polio among the departed UK-bound refugees, January 2018–October 2019 (N=6870)

		**Refugees in cohort**	**Refugee immunised in accordance with UK Refugee Technical Instructions (at least one dose; n=5798)**		**Refugee immunised in accordance with UK immunisation schedule (n=764)**	
			n (%)	Odds ratio (95% CI)	n (%)	Odds ratio (95% CI)
Sex						
	Female	3337	2794 (83·7%)	1 (ref)	370 (11·1%)	1 (ref)
	Male	3533	3004 (85·0%)	1·10 (0·96–1·26)	394 (11·2%)	0·92 (0·77–1·09)
Age						
	Child (<10 years)	2195	1936 (88·2%)	1 (ref)	706 (32·2%)	1 (ref)
	Adolescent (10–19 years)	1438	1190 (82·8%)	0·62 (0·50–0·75)	56 (3·9%)	0·09 (0·07–0·12)
	Adult (>19 years)	3237	2672 (82·5%)	0·59 (0·50–0·69)	2 (0·1%)	0·0013 (0·0001–0·0052)
Nationality*						
	Syrian	5032	4283 (85·1%)	1 (ref)	647 (12·9%)	1 (ref)
	Iraqi	353	329 (93·2%)	2·39 (1·56–3·65)	33 (9·3%)	0·81 (0·54–1·23)
	Sudanese	292	146 (50·0%)	0·17 (0·14–0·22)	34 (11·6%)	0·80 (0·53–1·20)
	Somalian	287	277 (96·5%)	0·52 (0·25–1·07)	23 (8·0%)	1·79 (0·76–4·23)
	Afghan	155	133 (85·8%)	0·92 (0·58–1·48)	0%	..
	Palestinian	144	126 (87·5%)	1·30 (0·79–2·15)	0%	..
	Congolese (DR Congo)	228	217 (95·2%)	0·07 (0·02–0·22)	10 (4·4%)	1·24 (0·40–3·85)
	Eritrean	156	111 (71·2%)	0·15 (0·10–0·23)	5 (3·2%)	0·48 (0·18–1·27)
Region of health assessment						
	Eastern Mediterranean	5561	4585 (82·5%)	1 (ref)	663 (11·9%)	1 (ref)
	Europe	669	590 (88·2%)	1·74 (1·33–2·27)	71 (10·6%)	1·36 (1·01–1·86)
	Africa	608	594 (97·7%)	59·41 (20·50–172·16)	28 (4·6%)	0·24 (0·10–0·59)

* Top eight nationalities only.

**Table 3:** Factors associated with being in line with the UK Refugee Technical Instructions (at least one dose) and being in accordance with the UK immunisation schedule for measles among the departed UK-bound refugees, January 2018–October 2019 (N=6870)

		**Refugees in cohort**	**Refugee immunised in accordance with UK Refugee Technical Instructions (at least one dose; n=5798)**		**Refugee immunised in accordance with UK immunisation schedule (n=764)**	
			n (%)	OR (95% CI)	n (%)	OR (95% CI)
Sex						
	Female	3337	2688 (80·6%)	1 (ref)	1090 (32·7%)	1 (ref)
	Male	3533	2868 (81·2%)	1·04 (0·92–1·18)	1248 (35·3%)	1·05 (0·94–1·17)
Age						
	Child (<10 years)	2195	1738 (79·2%)	1 (ref)	1118 (50·9%)	1 (ref)
	Adolescent (10–19 years)	1438	1181 (82·1%)	1·27 (1·06–1·53)	445 (31·9%)	0·41 (0·35–0·47)
	Adult (>19 years)	3237	2637 (81·5%)	1·14 (0·99–1·31)	775 (23·9%)	0·29 (0·25–0·32)
Nationality*						
	Syrian	5032	4109 (81·7%)	1 (ref)	1957 (38·9%)	1 (ref)
	Iraqi	353	322 (91·2%)	2·17 (1·49–3·16)	150 (42·5%)	1·13 (0·90–1·42)
	Sudanese	292	169 (57·9%)	0·32 (0·25–0·41)	62 (21·2%)	0·42 (0·32–0·57)
	Somalian	287	256 (89·2%)	0·17 (0·10–0·30)	43 (15·0%)	0·48 (0·26–0·88)
	Afghan	155	131 (84·5%)	0·86 (0·54–1·36)	38 (24·5%)	0·36 (0·24–0·54)
	Palestinian	144	106 (73·6%)	0·66 (0·44–0·96)	20 (13·9%)	0·27 (0·17–0·44)
	Congolese (DR Congo)	228	207 (90·8%)	0·08 (0·04–0·18)	17 (7·5%)	0·25 (0·11–0·54)
	Eritrean	156	86 (55·1%)	0·06 (0·04–0·10)	10 (6·4%)	0·12 (0·06–0·25)
Region of health assessment						
	Eastern Mediterranean	5561	4378 (78·7%)	1 (ref)	1923 (34·6%)	1 (ref)
	Europe	669	580 (86·7%)	1·99 (1·54–2·04)	353 (52·8%)	2·83 (2·36–3·40)
	Africa	608	566 (93·1%)	28·02 (15·61–50·28)	59 (9·7%)	0·53 (0·29–0·96)

* Top eight nationalities only.

The proportion of individuals who had departed to the UK with no recorded vaccinations varied by nationality and country of health assessment and for different diseases. Compared with the overall proportion in the database, Sudanese and Eritreans most frequently had no doses for polio (Sudanese 150 [51·4%] of 292; Eritrean 45 [28·8%] of 156), measles (Sudanese 123 [42·1%]; Eritrean 70 [44·9%]), or diphtheria/tetanus (Sudanese 264 [90·4%]; Eritrean 144 [92·3%]) on departure. When compared with the overall proportion, individuals who underwent health assessment in Turkey most frequently had at least one recorded dose of either measles (538 [96·8%] of 556), diphtheria, tetanus, and pertussis vaccine (505 [90·8%]) or polio-containing (542 [97·5%]) vaccines on departure, whereas those in Egypt were the least likely to have the same (measles 653 [53·5%] of 1220; diphtheria, tetanus, and pertussis vaccine 71 [6·2%]; and polio 588 [48·2%]).

We found that in terms of polio vaccination coverage, adolescents were around ten-times less likely than children to align with the UK immunisation schedule (odds ratio [OR] 0·09, 95% CI 0·07–0·12) and adults were considerably less likely to be aligned (0·0013, 0·0001–0·0052; table 2). Sudanese (OR 0·17, 95% CI 0·14–0·22), Congolese (0·07, 0·02–0·22), and Eritreans (0·15, 0·10–0·23) were significantly less likely to have received at least one polio dose, as per the UK Refugee Technical Instructions, compared with the reference group (Syrians). Compared to those assessed in the Eastern Mediterranean region, those assessed in the European region were significantly more likely to have at least one dose (OR 1·74, 95% CI 1·33–2·27) and be aligned with the UK schedule (1·36, 1·01–1·86) for polio-containing vaccine; those assessed in the African region were substantially more likely to have received at least one dose (59.·41, 20.·50–172.·16), but significantly less likely to be aligned with the UK immunisation schedule (0·24, 0·10–0·59; table 2).

Adults (OR 0·29, 95% CI 0·25–0·32) and adolescents (0·41, 0·35–0·47) were significantly less likely to be vaccinated to the UK immunisation schedule for measles than were children, and adolescents were statistically more likely to have at least one dose. Compared with Syrians, Iraqis were most likely to have at least one dose of measles vaccination (2·17, 1·49–3·16; table 3). Individuals based in the European region were significantly more likely to align with the UK Refugee Technical Instructions (OR 1·99, 95% CI 1·54–2·04) and the UK immunisation schedule (2·83, 2·36–3·40) compared with individuals based in the Eastern Mediterranean region, whereas those in the African region were more likely (28·04, 15·61–50·28) to have at least one dose of measles-containing vaccine and were less likely to have vaccination in line with the UK immunisation schedule (table 3).

## Discussion

This study examined International Organization for Migration's vaccination activities during pre-departure migrant health assessments under the UK refugee resettlement programme and the subsequent immunisation coverage of 12 526 UK-bound refugees undergoing an migration health assessment. 26 118 vaccine doses were given by the IOM across 18 countries (appendix pp 3–4), aligning with the UK Refugee Technical Instructions guidelines, which focus on providing at least one dose of measles-containing and polio-containing vaccine.

Although the UK Refugee Technical Instructions were acted upon in most cases, we show that more than one in ten (15·6%) refugees departed for the UK with no recorded polio-containing vaccine, and almost one in five (19·1%) without a recorded measles-containing vaccine. The UK Refugee Technical Instructions does not give guidance on diphtheria-containing and tetanus-containing vaccines, and around four in five (80·9%) refugees arrived in the UK without a recorded dose. Adults and adolescents were less likely than children to align with the UK Refugee Technical Instructions and the UK immunisation schedule for polio. Sudanese and Eritrean nationals most frequently had no doses for polio and measles (tables 2, 3). Many UK-bound resettled refugees, particularly adults and adolescents, will need appropriate catch up vaccination services on arrival, in line with recommendations in the WHO Immunization Agenda 2030 to ensure equitable vaccination coverage across the life course.^29^

We have shown that the immunisation coverage of UK-bound refugees varied by country of health assessment, often tethered to distinct refugee populations. Variations by nationality were also reflected in the data. Sudanese and Eritrean nationals had low reported coverages for key vaccines, whereas Iraqi nationals had comparatively high recorded coverages in this cohort. The reasons for varying vaccination yields are multifactorial; some determinants stem from vaccine availability, country-level national policy and the UK Refugee Technical Instructions, which only provide specific guidelines for measles and polio and do not align with the UK schedule. The International Organization for Migration's capacity to deliver vaccination is determined by these factors and funding required to procure vaccines. The IOM only administers vaccines that have been registered in-country and approved by the national ministries of health to use on populations under their jurisdiction. Furthermore, vaccine procurement is done through national government programmes therefore the IOM is dependent on external allocations, funding, and supply chains.

Although the UK Government funds the UK refugee health assessment programme, unlike the comprehensive premigration immunisation programme for refugees resettling in the USA.^30^ There is no contractual obligation for the UK Refugee Technical Instructions to be followed in full for a refugee to enter the UK. This factor could account for the differences in outcomes, with the US programme resulting in coverage rates of more than 90% for polio, measles, and the diphtheria, tetanus, and pertussis vaccine.^30^ There is little published data on pre-departure vaccination campaigns from other countries of resettlement; however, data from Canada shows that resettled refugees have significantly higher vaccine-preventable disease-related hospitalisation rates than Canadian born individuals, suggesting they could be underimmunised.^31^ However, when comparing UK pre-migration health programmes to those of other countries, we should bear in mind that NHS vaccinations are free at point-of-care in the UK after arrival, regardless of immigration status, including through a childhood and an adult immunisation programme, and vaccination for individuals with incomplete or unknown immunisation status. Guidance from the UK Health Security Agency clearly states that “if children and adults coming to the UK do not have a documented or reliable history of immunisation, they should be assumed to be unimmunised and required immunisations planned”.^26^

Immunisation levels varied between age groups in our data, with adolescents and adults less likely to have key vaccines recorded, suggesting that they merit greater focus in the International Organization for Migration health assessments, but also on arrival to the UK. Considering that children are easily incorporated into the UK immunisation schedule through the British school system, these data highlight the importance of catch-up immunisation for adults, which currently has major shortfalls.^32^ New approaches might need to be developed, such as improvement of the data flows between the IOM and UK primary care services. It is essential that primary care services in areas receiving resettled refugees are aware of the importance of adult catch-up vaccination and that refugees are included in local needs assessments. The low coverage found for vaccinations recommended (meningococcal, *H influenzae*, varicella, and rotavirus vaccines) by the UK Refugee Technical Instructions to be given in crowded conditions should also be considered, as outbreaks of these diseases have occurred in the past two decades in refugees in Europe.^8^, ^9^, ^33^ These vaccinations are often not covered by national schedules, suggesting increased emphasis could be put on these vaccines as part of pre-departure vaccination programmes or novel pathways developed to deliver them to high-risk populations on arrival.

The extent to which these findings on immunisation coverage in UK-bound refugees are relevant to other migrant groups, such as undocumented migrants, asylum seekers, and labour migrants, entering the UK (and other high-income countries) is particularly pertinent during the ongoing COVID-19 vaccination rollouts and merits urgent further research. We have shown in a systematic review that refugees and migrants could be an at-risk group for vaccine-preventable disease outbreaks in Europe,^8^ and low immunisation coverage in refugees and migrants to the EU has previously been highlighted.^34^ In the UK setting, this is in the context of declining vaccination uptake in the wider population in recent years; in England in 2018–19, coverage declined in all routine vaccinations compared with the previous year, including for diphtheria, tetanus, pertussis, polio, *H influenzae*, measles, mumps, and rubella.^35^

Going forward, greater definition might be required on where the responsibility lies for providing vaccination to resettled refugees. Processes to ensure effective links to primary care services will be needed if host countries are to provide catch-up vaccines for adolescent and adult refugees, and other migrants. These services should be evidence-based and consider previous vaccinations, including those received from the IOM for resettled refugees, to prevent duplication of efforts and over-vaccination. This process will require collaboration between public (the UK Health Security Agency and the Home Office) and academic bodies to synthesise all existing data on immunisation coverage among refugees and improve data collection pathways. Further research should also be carried out on vaccine uptake and demand issues (including confidence, convenience, and complacency factors) in specific migrant groups, such as resettled refugees on arrival to the UK,^36^ as concerns have arisen during COVID-19 campaigns that some refugee and migrant groups are facing barriers to access or a reluctance to vaccinate.^37^, ^38^, ^39^ This hesitancy could require greater focus on co-designing strategies in close collaboration with affected communities.^37^ Views of resettled refugees taking part in the UK refugee vaccination programme should be sought to assess acceptability of vaccination services and areas for improvement pre-arrival and post-arrival.

There is likely to have been underestimation of immunisation coverage before presenting at migration health assessments as often refugees in the resettlement programme do not have vaccine records on them, as most individuals are assessed outside their country of origin after fleeing war or persecution. Therefore, these records are not routinely asked for during migration health assessments. Despite this gap in historical vaccine data, our findings have important policy and practice implications for immunisation as part of resettlement programmes and catch-up vaccinations on arrival to the UK, where those missing vaccination records will be treated as unvaccinated as per the algorithm for vaccination of individuals with uncertain or incomplete immunisation status.^27^ Although this study used at least one recorded dose as a proxy for being in line with the UK Refugee Technical Instructions for polio and measles, our analysis did not consider factors resulting in ineligibility for vaccines, such as being aged younger than 9 months (n<1 year old=266) or HIV positive status (n=14). These limitations could produce some bias; however, being aged younger than 9 months can be assumed (data only available for <1 year olds) to be by far the biggest of these categories, representing around 10% of children who had departed for the UK. Nevertheless, children were still shown to be significantly more likely to be vaccinated for measles, suggesting this relationship would retain the same directionality if ineligible individuals could be excluded.

Our findings have important policy and practice implications for the vaccination of refugees arriving in high-income countries, including in ongoing COVID-19 vaccine rollouts. Refugees with missing records of key vaccines will be considered to be under-immunised in terms of UK policy and will require routine catch-up vaccinations on arrival through UK primary care. A focus is required on adults and adolescents who might not be easily incorporated into immunisation services. These data have shed light on the shortfalls in the vaccination of refugees and can be used to inform approaches to catch-up vaccination for refugees arriving in the UK and other high-income countries, with implications for strategies in wider migrant groups, which are key focuses of the WHO Immunization Agenda 2030.

## Data sharing

Abridged versions (to maintain full anonymity) of the datasets used and analysed during the study are available from the corresponding author on reasonable request.

## Declaration of interests

SH is a freelance Senior Editor for *The Lancet Infectious Diseases* and other *Lancet* journals. AD and SEH are funded by the Medical Research Council (MR/N013638/1). SH is funded by the National Institute for Health and Care Research (NIHR; NIHR Advanced Fellowship NIHR300072), the Academy of Medical Sciences (SBF005\1111), the Novo Nordisk Foundation/La Caixa Foundation (Mobility–Global Medicine and Health Research grant), and the WHO. AFC is funded by the NIHR (NIHR Advanced Fellowship NIHR300072) and the Academy of Medical Sciences (SBF005\1111). LPG is funded by the NIHR (NIHR300072). All other authors declare no competing interests. The views expressed are those of the authors and not necessarily those of the NHS, the NIHR, or the Department of Health and Social Care.
