# Supplementary material for: Immunisation status of UK-bound refugees between January, 2018, and October, 2019: a retrospective, population-based cross-sectional study
Source: Lancet Public Health. 2022 May 28;7(7):e606–15. doi: 10.1016/S2468-2667(22)00089-5 (PMC9581781; doi:10.1016/S2468-2667(22)00089-5)
Supplement: Supplementary appendix [file mmc1.pdf]

THE LANCET
Public Health

## Supplementary appendix

This appendix formed part of the original submission and has been peer reviewed. We post it as supplied by the authors.

Supplement to: Deal A, Hayward SE, Crawshaw AF, et al. Immunisation status of UK-bound refugees between January, 2018, and October, 2019: a retrospective, population-based cross-sectional study. *Lancet Public Health* 2022; published online May 27. https://doi.org/10.1016/S2468-2667(22)00089-5.

**Appendix**

**Supplementary Table 1: Description of the UK refugee technical instructions (UKREF TI) and the UK immunisation schedule for key diseases**

| Disease | UKREF TI | UK immunisation schedule |
|---|---|---|
| **Polio** | At least one dose of oral polio vaccine (OPV) or inactivated polio vaccine (IPV), depending on the type of circulating polio and vaccine availability | 2 months – 3 years: 3 doses, Age 3+ years: 4 doses |
| **Measles** | At least one dose, preferably measles, mumps and rubella (MMR) to all refugees older than 9 months | 1-3 years :1 dose,<br>Age 3+ years: 2 doses |
| **Mumps** | N/A | 1-3 years old: 1 dose<br>3+ years old: 2 doses |
| **Rubella** | N/A | 1-3 years: 1 dose,<br>3+ years old: 2 doses |
| **Diphtheria/Tetanus** | N/A | 2 months – 3 years: 3 doses,<br>3+ years old: 4 doses |
| **Pertussis** | N/A | 2 months – 3 years: 3 doses<br>3 years old: 4 doses |

| | | |
|---|---|---|
| **Hepatitis B** | To close contacts of identified cases | 2 months+: 3 doses |
| **Rotavirus** | Advised if living in crowded conditions | 2 months+: 2 doses |
| **Varicella** | Advised if living in crowded conditions | NA |
| **Meningococcal** | Advised if living in crowded conditions | MenB/2 months-1 year: 3 doses,<br><br>MenC/1+ years: 1 dose,<br><br>MenACWY/14+ years: 1 dose |
| **Pneumococcal** | N/A | 2 months – 1 year: 2 doses |
| **Hib** | Advised if living in crowded conditions | 2 months – 1 year: 3 doses |

**Appendix Supplementary Table 2.** Count of doses given during IOM migration health assessments, by vaccine type, region and country of health assessment and age category.

| | IPV | OPV | MMR | Measles | Mumps | Rubella | Td | DTaP | Tdap | DTP | HepB | Meningococcal | Hexavalent | Hib | Varicella | Influenza | PCV13 | Other MCV | Pentacel | All vaccines |
|---|---|---|---|---|---|---|---|---|---|---|---|---|---|---|---|---|---|---|---|---|
| Total doses | 1549 | 8251 | 8741 | 243 | 3 | 2 | 46 | 7 | 1143 | 84 | 4529 | 1429 | 44 | 3 | 9 | 28 | 1 | 5 | 1 | 26118 |
| **Region/Country of health assessment** | | | | | | | | | | | | | | | | | | | | |
| **Eastern Med** | **89** | **7427** | **6199** | **241** | **1** | **0** | **0** | **0** | **0** | **84** | **3717** | **759** | **1** | **1** | **0** | **2** | **0** | **0** | **0** | **18521** |
| Afghanistan | 0 | 1 | 0 | 0 | 0 | 0 | 0 | 0 | 0 | 0 | 0 | 0 | 0 | 0 | 0 | 0 | 0 | 0 | 0 | 1 |
| Syria | 0 | 255 | 0 | 0 | 0 | 0 | 0 | 0 | 0 | 0 | 2 | 0 | 0 | 0 | 0 | 0 | 0 | 0 | 0 | 257 |
| Egypt | 0 | 1068 | 727 | 0 | 0 | 0 | 0 | 0 | 0 | 0 | 1236 | 710 | 0 | 0 | 0 | 0 | 0 | 0 | 0 | 3741 |
| Sudan | 23 | 0 | 0 | 0 | 0 | 0 | 0 | 0 | 0 | 0 | 1 | 0 | 0 | 0 | 0 | 0 | 0 | 0 | 0 | 24 |
| Lebanon | 66 | 3305 | 3233 | 0 | 0 | 0 | 0 | 0 | 0 | 0 | 1594 | 9 | 0 | 0 | 0 | 0 | 0 | 0 | 0 | 8207 |
| Iran | 0 | 197 | 198 | 0 | 1 | 0 | 0 | 0 | 0 | 0 | 119 | 0 | 0 | 0 | 0 | 0 | 0 | 0 | 0 | 515 |
| Iraq | 0 | 1242 | 1178 | 0 | 0 | 0 | 0 | 0 | 0 | 2 | 186 | 11 | 1 | 1 | 0 | 2 | 0 | 0 | 0 | 2623 |
| Jordan | 0 | 1359 | 863 | 241 | 0 | 0 | 0 | 0 | 0 | 82 | 579 | 29 | 0 | 0 | 0 | 0 | 0 | 0 | 0 | 3153 |
| **Europe** | **1197** | **0** | **1465** | **0** | **0** | **0** | **29** | **7** | **1143** | **0** | **429** | **655** | **36** | **2** | **0** | **0** | **0** | **0** | **1** | **4964** |
| Greece | 82 | 0 | 39 | 0 | 0 | 0 | 29 | 1 | 51 | 0 | 0 | 60 | 1 | 0 | 0 | 0 | 0 | 0 | 0 | 263 |
| Turkey | 1115 | 0 | 1426 | 0 | 0 | 0 | 0 | 6 | 1092 | 0 | 429 | 595 | 35 | 2 | 0 | 0 | 0 | 0 | 1 | 4701 |
| **Africa** | **248** | **789** | **993** | **2** | **2** | **2** | **0** | **0** | **0** | **0** | **379** | **0** | **0** | **0** | **1** | **1** | **0** | **0** | **0** | **2417** |
| Niger | 0 | 161 | 153 | 0 | 0 | 0 | 0 | 0 | 0 | 0 | 72 | 0 | 0 | 0 | 1 | 0 | 0 | 0 | 0 | 387 |
| Kenya | 243 | 474 | 686 | 0 | 2 | 0 | 0 | 0 | 0 | 0 | 279 | 0 | 0 | 0 | 0 | 1 | 0 | 0 | 0 | 1685 |
| Burundi | 5 | 154 | 154 | 2 | 0 | 2 | 0 | 0 | 0 | 0 | 28 | 0 | 0 | 0 | 0 | 0 | 0 | 0 | 0 | 345 |
| **Asia** | **0** | **35** | **60** | **0** | **0** | **0** | **0** | **0** | **0** | **0** | **3** | **13** | **0** | **0** | **0** | **0** | **0** | **0** | **0** | **111** |
| Sri Lanka | 0 | 8 | 6 | 0 | 0 | 0 | 0 | 0 | 0 | 0 | 0 | 0 | 0 | 0 | 0 | 0 | 0 | 0 | 0 | 14 |

| | | | | | | | | | | | | | | | | | | | | |
|---|---|---|---|---|---|---|---|---|---|---|---|---|---|---|---|---|---|---|---|---|
| Thailand | 0 | 22 | 29 | 0 | 0 | 0 | 0 | 0 | 0 | 0 | 0 | 0 | 0 | 0 | 0 | 0 | 0 | 0 | 0 | 51 |
| India | 0 | 5 | 25 | 0 | 0 | 0 | 0 | 0 | 0 | 0 | 3 | 13 | 0 | 0 | 0 | 0 | 0 | 0 | 0 | 46 |
| **Pacific** | **15** | **0** | **24** | **0** | **0** | **0** | **17** | **0** | **0** | **0** | **1** | **2** | **7** | **0** | **8** | **25** | **1** | **5** | **0** | **105** |
| Malaysia | 15 | 0 | 24 | 0 | 0 | 0 | 17 | 0 | 0 | 0 | 1 | 2 | 7 | 0 | 8 | 25 | 1 | 5 | 0 | 105 |
| **Age range** | | | | | | | | | | | | | | | | | | | | |
| **Child (<10 years)** | **310** | **2124** | **2103** | **45** | **2** | **1** | **0** | **3** | **199** | **84** | **2213** | **621** | **43** | **3** | **4** | **11** | **1** | **0** | **1** | 7768 |
| **Adolescent (10-19 years)** | **444** | **1953** | **2134** | **63** | **0** | **0** | **36** | **1** | **346** | **0** | **2135** | **646** | **1** | **0** | **5** | **7** | **0** | **5** | **0** | 7776 |
| **Adult >19 years)** | **795** | **4174** | **4504** | **135** | **1** | **1** | **10** | **3** | **598** | **0** | **181** | **162** | **0** | **0** | **0** | **10** | **0** | **0** | **0** | 10574 |

Definitions: IPV: Inactivated polio vaccine, OPV: oral polio vaccine, MMR: measles-mumps-rubella, Td: Tetanus-diphtheria, DTaP/DTP/Tdap: tetanus-diphtheria-pertussis, HepB: hepatitis B, Hexavalent: diphtheria-tetanus-pertussis-polio-hepatitis B-Hib, Hib: *Haemophilus influenzae B*, PCV13: pneumococcus, Other MCV: meningococcus, Pentacel: diphtheria-tetanus-pertussis-polio-Hib

**Appendix Supplementary Table 3. Count of refugees immunised with at least one dose of key vaccines, by age category and region of health assessment**

| Characteristic | N in cohort | Refugees immunised with at least one dose, n (%) | | | | | | | | | | | |
|---|---|---|---|---|---|---|---|---|---|---|---|---|---|
| | | Polio | Measles | Mumps | Rubella | Diphtheria-tetanus | Pertussis | Hepatitis B | Rotavirus | Varicella | Meningococcus | Pneumococcus | Hib |
| **Total** | 6870 | 5798 (84.4) | 5556 (80.9) | 5485 (79.8) | 5484 (79.8) | 1315 (19.1) | 1193 (17.4) | 2809 (40.9) | 186 (2.7) | 32 (0.5) | 726 (10.6) | 98 (1.4) | 527 (7.7) |
| **Age** | | | | | | | | | | | | | |
| Child (<10) | 2195 | 1936 (88.2) | 1738 (89.8) | 1705 (88.1) | 1704 (88.0) | 758 (39.2) | 753 (38.9) | 1717 (88.7) | 186 (9.6) | 27 (1.4) | 308 (15.9) | 96 (5.0) | 488 (25.2) |
| Adolescent (10-19) | 1438 | 1190 (82.8) | 1181 (82.1) | 1163 (80.9) | 1163 (80.9) | 241 (16.8) | 183 (12.7) | 1000 (69.5) | 0 (0) | 5 (0.3) | 322 (22.4) | 0 (0) | 36 (2.5) |
| Adult (>19) | 3237 | 2672 (82.6) | 2637 (81.5) | 2617 (80.8) | 2617 (80.8) | 316 (9.8) | 257 (7.9) | 92 (2.8) | 0 (0) | 0 (0) | 96 (3.0) | 2 (0.06) | 3 (0.09) |
| **Region of Health Assessment** | | | | | | | | | | | | | |
| Eastern Mediterranean | 5561 | 4585 (82.5) | 4378 (78.7) | 4306 (77.4) | 4306 (77.4) | 719 (12.9) | 626 (11.3) | 2386 (42.9) | 161 (2.9) | 4 (0.07) | 385 (6.9) | 57 (1.0) | 410 (7.4) |
| Europe | 669 | 590 (88.2) | 580 (86.7) | 580 (86.7) | 580 (86.7) | 552 (82.5) | 533 (79.7) | 216 (32.3) | 1 (0.1) | 24 (3.6) | 338 (50.5) | 9 (1.3) | 83 (12.4) |
| Africa | 608 | 594 (97.7) | 566 (93.1) | 567 (93.3) | 566 (93.1) | 28 (4.6) | 28 (4.6) | 200 (32.9) | 24 (3.9) | 1 (0.2) | 0 (0) | 31 (5.1) | 28 (4.6) |
| Asia | 15 | 15 (100) | 15 (100) | 15 (100) | 15 (100) | 0 (0) | 0 () | 0 () | 0 (0) | 0 (0) | 0 (0) | 0 (0) | 0 (0) |
| Pacific | 17 | 14 (82.4) | 17 (100) | 17 (100) | 17 (100) | 16 (94.1) | 6 (35.3) | 7 (41.2) | 0 (0) | 3 (17.6) | 3 (17.6) | 1 (5.9) | 6 (35.3) |

**Appendix Supplementary Table 4. Count of refugees immunised according to the UK schedule, by age category and region of health assessment**

| Characteristic | N in cohort | Refugees immunised according to the UK schedule, n (%) | | | | | | | | | | | |
|---|---|---|---|---|---|---|---|---|---|---|---|---|---|
| | | Polio | Measles | Mumps | Rubella | Diphtheria-tetanus | Pertussis | Hepatitis B | Rotavirus | Varicella[1] | Meningococcus[2] | Pneumococcus | Hib |
| **Total** | 6870 | 764 (11.1) | 2338 (34.0) | 2217 (32.3) | 2217 (32.3) | 380 (5.5) | 375 (5.5) | 668 (9.7) | 167 (2.6) | - | - | 75 (1.2) | 430 (6.3) |
| **Age** | | | | | | | | | | | | | |
| Child (<10) | 2195 | 706 (32.2) | 1118 (50.9) | 1055 (48.1) | 1055 (48.1) | 349(15.9) | 346 (15.8) | 576 (26.2) | 167 (7.6) | - | - | 75 (3.4) | 345 (15.7) |
| Adolescent (10-19) | 1438 | 56 (3.9) | 445 (30.9) | 403 (28.2) | 403 (28.2) | 30(2.1) | 29 (2.0) | 74 (5.1) | 0 (0) | - | - | 0 (0) | 26 (1.8) |
| Adult (>19) | 3237 | 2 (0.06) | 775 (23.9) | 759 (23.4) | 759 (23.4) | 1(0.03) | 0 (0) | 18 (5.6) | 0 (0) | - | - | 0 (0) | 0 (0) |
| **Region of Health Assessment** | | | | | | | | | | | | | |
| Eastern Mediterranean | 5561 | 663 (11.9) | 1923 (34.6) | 1810 (32.5) | 1810 (32.5) | 309 (5.6) | 305 (5.5) | 588 (10.6) | 148 (2.7) | - | - | 45 (0.8) | 345 (6.2) |
| Europe | 669 | 71 (10.6) | 353 (52.8) | 352 (52.6) | 352 (52.6) | 55 (8.2) | 54 (8.1) | 56 (8.4) | 1 (0.1) | - | - | 4 (0.6) | 60 (9.0) |
| Africa | 608 | 28 (4.6) | 59 (9.7) | 52 (8.6) | 52 (8.6) | 14 (2.3) | 14 (2.3) | 22 (3.6) | 18 (3.0) | - | - | 26 (4.3) | 23 (3.8) |
| Asia | 15 | 0 (0) | 0 (0) | 0 () | 0 (0) | 0 (0) | 0 (0) | 0 (0) | 0 (0) | - | - | 0 (0) | 0 (0) |
| Pacific | 17 | 2 (11.8) | 3 (17.6) | 3 (17.6) | 3 (17.6) | 2 (11.8) | 2 (11.8) | 2 (11.8) | 0 (0) | - | - | 0 (0) | 2 (11.8) |
